# Evaluation of Dynamic Respiratory Muscle Strength, Physical Fitness, and Physical Activity in Children with Asthma and Healthy Peers

**DOI:** 10.3390/healthcare12242579

**Published:** 2024-12-21

**Authors:** Seyma Nur Onal, Gulnar Aliyeva, Ebru Calik Kutukcu, Naciye Vardar Yagli, Bulent Enis Sekerel, Ozge Uysal Soyer, Umit Murat Sahiner

**Affiliations:** 1 Physiotherapy Program, Department of Therapy and Rehabilitation, Vocational School of Health Services, Bartın University, 74110 Bartin, Turkey; 2 Department of Pediatric Allergy, Hacettepe University School of Medicine, 06230 Ankara, Turkey; gulnaraliyeva2016@hotmail.com (G.A.); b_sekerel@yahoo.com (B.E.S.); ozgeusoyer@gmail.com (O.U.S.); umsahner@yahoo.com (U.M.S.); 3 Department of Cardiorespiratory Physiotherapy and Rehabilitation, Faculty of Physical Therapy and Rehabilitation, Hacettepe University, 06230 Ankara, Turkey; ebrucalk85@hotmail.com (E.C.K.); naciyevardar@yahoo.com (N.V.Y.)

**Keywords:** asthma, child, S-index, fitness, physical activity

## Abstract

**Background**: Systemic inflammation, attacks, deterioration of thoracic region mechanics, medications used, and decreased physical activity level (PAL) and fitness negatively may affect respiratory muscle strength. The primary aim of this study was to evaluate dynamic inspiratory muscle strength (S-index), PAL, and physical fitness in children with asthma compared to healthy peers. The secondary aim of this study was to investigate the relationships between S-index and peak inspiratory flow (PIF) values and functional parameters in childhood asthma. **Methods**: This cross-sectional prospective study consisted of participants of 6–11 years of age, specifically, 20 children with asthma and 20 healthy peers. The device (POWERbreathe K5) evaluated PIF and S-index variables. PAL was determined with the Physical Activity Questionnaire-Child (PAQ-C) and fitness was evaluated with the FITNESSGRAM test battery. **Results**: The PIF Average (Avg): 1.8 ± 0.6, Best: 2.6 ± 0.6 (asthmatic children) vs. Avg: 2.4 ± 0.7, Best: 3.1 ± 0.9 (healthy children); *p* = 0.017, *p* = 0.027, respectively) and S-index (Avg: 34.1 ± 10.3, Best: 45.6 ± 9.9 (asthmatic children) vs. Avg: 43.2 ± 12.1, Best: 56.6 ± 14.7 (healthy children); *p* = 0.015, *p* = 0.008 respectively) values of children with asthma were significantly lower compared to healthy peers. The PAL and physical fitness of asthmatic and healthy peers were similar (*p* > 0.05). There were significant relationships between S-indexavg and S-indexbest with the PAQ-C score (r = 0.498, *p* < 0.025 r = 0.547, *p* < 0.013, respectively) and PIFavg and PIFbest with the PAQ-C score (r = 0.490, *p* < 0.028 r = 0.602, *p* < 0.005) in children with asthma. **Conclusions**: Dynamic respiratory muscle strength is negatively affected in children with asthma whereas their physical activity and fitness levels are comparable to their peers. However, it was found that children with a higher S-index and PIF rate had higher PALs. These findings suggest that pulmonary rehabilitation interventions to improve respiratory muscle function should be considered an important strategy to maintain and increase physical activity levels in children with asthma.

## 1. Introduction

Asthma is the most common respiratory disease among children worldwide [1]. Asthma is characterized by inflammation of the airways, bronchoconstriction (airway narrowing), and temporary airway obstruction [2,3]. Respiratory muscles weaken as a result of oxidative stress, hypoxia, and chronic systemic inflammation throughout the pathophysiologic processes of asthma [4]. The load created by airway obstruction, especially on inspiratory muscles; hyperinflation caused by obstruction of expiratory airflow during attacks; and changes in the length–tension relationship of the diaphragm may reduce respiratory muscle strength in children with asthma [5]. Preserved respiratory muscle strength is so important for better respiratory functions, decreased inflammation biomarkers, and asthma symptoms in children with asthma [6,7]. Therefore, the strength of the respiratory muscles in children with asthma is recognized as an important parameter in the management and treatment of the disease [8].

Respiratory and skeletal muscle strength, exercise capacity and physical activity levels (PALs) are generally reduced in children with asthma [9,10,11]. Respiratory restrictions and symptoms during exercise can limit children’s participation in physical activity, lead to muscle weakness, and negatively affect the course of the disease [12,13]. Low PALs can lead to weakened respiratory muscles, worsening the overall health of children with asthma [14]. However, some studies show that children with asthma have similar respiratory muscle strength to their healthy peers [15,16]. These conflicting results call for a more detailed examination of the effects of asthma.

Although there are many studies in the literature examining the relationship between respiratory muscle strength, physical activity, and exercise capacity in children with asthma, these studies are generally limited to static pulmonary function measurements [9,17]. For example, while measurements such as peak inspiratory flow (PIF) and forced expiratory volume in one second (FEV_1_) are widely used, research on dynamic pulmonary function is very limited [18,19]. The advantage of dynamic measurement in asthmatic children is that it can assess the strength of the respiratory muscles not only isometrically (statically) but also in full amplitude throughout the entire chest movement. Dynamic measurement can record changes in muscle strength during inspiration and offers more precise results through graphical analysis [20]. Furthermore, a better understanding of the relationships between dynamic inspiratory muscle strength (S-index) and the PAL and exercise capacity in children with asthma may help to develop more effective approaches to asthma treatment [21].

There are also conflicting results on limited data about physical fitness parameters. Cardiopulmonary and physical fitness levels are significantly lower or preserved in children with asthma compared to healthy children [22,23,24,25]. To the best of our knowledge, there is no study that interprets the S-index along with physical fitness and PAL measures in children with asthma. Therefore, the primary aim of this study was to evaluate dynamic inspiratory muscle strength, PALs, and physical fitness in children with asthma compared to healthy peers. The secondary aim of this study was to investigate the relationships between S-index and PIF values and functional parameters in patients with childhood asthma.

## 2. Material and Methods

### 2.1. Study Design

This study was conducted between December 2023 and March 2024 in Hacettepe University, Faculty of Physical Therapy and Rehabilitation, Department of Cardiorespiratory Physiotherapy and Rehabilitation. The Hacettepe University Ethics Committee approved the study committee (17 May 2023, with approval number GO 23/241) and this was registered in the National Institutes of Health Clinical Trial Institute, Identifier: NCT06053905 (https://clinicaltrials.gov/study/NCT06053905) (accessed on 17 December 2024).

### 2.2. Participants

**Figure 1:**
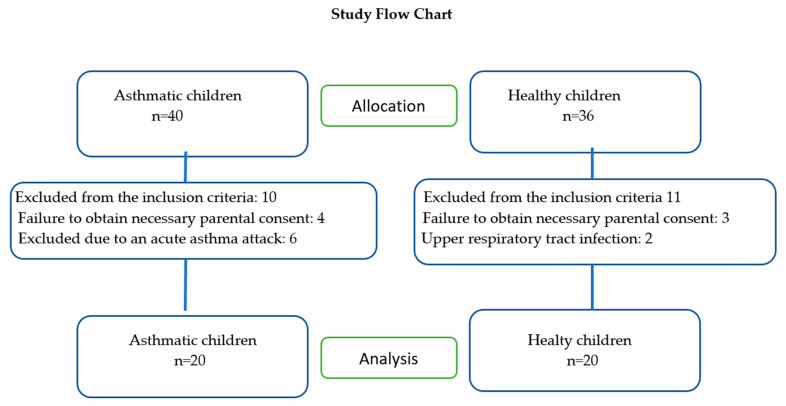
Study Flow Chart.

In this cross-sectional study, 20 asthmatic children under routine follow-up directed by the Hacettepe University Department of Pediatrics, Allergy and Asthma Unit, and 20 healthy children who volunteered to participate were included (Figure 1). The inclusion criteria for asthmatic children were that they be 6–11 years of age, diagnosed with childhood asthma based on Global Initiative for Asthma (GINA) guideline diagnostic criteria [26], clinically stable and not in an active asthma attack, approved for informed consent signed by parents or legal guardians, and cooperative with tests. The exclusion criteria for the patient group were having an asthma attack or being admitted or hospitalized in the last three months due to an asthma attack; serious diseases, such as cancers, heart failure, acute respiratory tract infections, chronic lung disease other than asthma, and severe neuromuscular and musculoskeletal problems; and being uncooperative to participate in the tests. The inclusion criteria for healthy children were that they be 6–11 years of age; without any chronic disease or a mental, neurological, orthopedic, cardiovascular, or pulmonary problem that would prevent physical activity; cooperative; able to volunteer to participate in this study; and able to have the free and informed consent form signed by their parents or legal guardians to allow the child to participate in this study.

### 2.3. Outcome Measures

The sociodemographic details of the participants with the clinical characteristics related to the disease (COVID-19, exercise intolerance, allergy history, colds reaching the lungs, and colds lasting longer than 10 days) were recorded. The body weight and height of children were measured with a portable stadiometer (Wunder Sa.Bi., RB200 (WU150), Trezzo sull’Adda, Italy). Body mass index (BMI) was calculated with the formula body weight/(height)^2^ and recorded as kg/m^2^.

#### 2.3.1. Dynamic Inspiratory Muscle Strength Test

The POWERbreathe K5 (HaB International Ltd., Southam, UK) smart digital respiratory muscle testing and training device was used for the S-index. The initial two breaths are taken rapidly and deeply to determine the maximum respiratory capacity. The loading resistance varies intermittently based on the breathing resistance and the device offers graphic feedback on the breathing pattern displayed on the screen. The device is concluded to ‘automatically process and provide valid estimates of physical energy units during loaded respiratory tasks’. It assesses based on S-index and PIF values. The device provides us with information on important parameters, such as load (cmH_2_O), power (Watt), volume (L), flow (L/sn.), and energy (Joule). The device also directly gives the evaluation results as average (‘avg’) and best performance (‘best’) data for all parameters. The device automatically calculates and records these metrics based on the participants’ performance. A nose clip, which comes with the POWERbreathe K5 device, was used during all uses to eliminate airflow outside the testing device. The POWERbreathe K5 was chosen for its ability to provide objective and reliable measurements of respiratory muscle performance both in the healthy population and patients with chronic diseases, with dynamic resistance adjustment and real-time feedback enhancing accuracy and participant engagement. Additionally, the determination of the S-Index provides valuable information about inspiratory muscle capacity [27].

#### 2.3.2. Physical Fitness Level

Fitness level was assessed using the FITNESSGRAM test battery [28]. FITNESSGRAM is a scientifically validated fitness assessment tool, with established reliability and validity for evaluating various physical fitness components, including cardiovascular endurance, muscular strength, and flexibility [23,29]:

(a) Back-Saver Sit and Reach Test: While sitting, the child stretched one leg and leaned it against a bench, bending the other lower limb at the knee. He reached forward four times, hands on top of each other. During the last stretch, the distance from the tip of the foot to the tip of the finger was measured on the ruler on the bench;
(b) Trunk Lift Test: The patient was placed in a prone position with his hands under his legs. He was told to lift his body twice, looking at the coin or sign. In the second attempt, the distance between the chin and the cushion was measured with a tape measure;
(c) Curl-Up Test: The child lay supine with flexed knees, arms at the sides on a 3-inch fixed paper, and bent his body forward in a rhythmic pace (3 s) while tracking the paper with the fingertips. The test ended when the maximum number of 75 was reached, an error occurred in paper tracking, or when they could no longer continue. The number of correctly performed repetitions was noted;
(d) Push-Up Test: The individual was placed face down with his hands at shoulder width. The upper body moved up and down (every three seconds) with the elbows bending and extending. The test was halted upon a second error, such as knees touching the ground, loss of back straightness, arms not fully extending, or the elbows not bending up to 90 degrees. The number of repetitions was documented;
(e) Progressive Aerobic Cardiovascular Endurance Run (PACER) Test: The test entailed running at an accelerating pace (decreasing by half a second every minute) on a 20-meter course. The test concluded if the pace was not maintained or if the finish line could not be reached before two beeps. The number of completed laps was recorded [30]. Shortness of breath, overall fatigue, and leg fatigue were assessed and recorded before and after the test. Participants’ maximum oxygen consumption (VO_2_max) values were calculated [31,32].

#### 2.3.3. Physical Activity Level

The Physical Activity Questionnaire for Older Children (PAQ-C) is a self-administered, 7-day recall questionnaire that assesses activities in physical activities (PAs) [33,34]. The scale consists of 9 questions; the 10th question asks whether there is any disease that prevents PA. The total score was divided by the number of questions. The total scores range between 1 and 5. As the score increases, the PAL increases [34].

### 2.4. Statistical Analysis

The statistical analyses were performed using SPSS Version 26.0 (IBM Corp., Armonk, NY, USA). Normal distribution of the variables was tested using histograms, Q–Q plots, and Shapiro–Wilk tests. The numerical data for the demographic and clinical features were presented as mean and standard deviation for normally distributed data. Otherwise, the median (IQR) was presented as descriptive statistics. Categorical data were represented as frequency (n) and percentage. The Chi-square test was used to compare categorical data. The parameters of the PAQ-C, FITNESSGRAM and Dynamic Inspiratory Muscle Strength Test were found to be normally distributed, except for Energytotal (Joule) and Energybest (Joule). However, the results of the PACER test did not conform to a normal distribution. An independent sample *t*-test was used to compare normally distributed numerical data of groups and a Mann–Whitney U test was used to compare non-normally distributed numerical data. The correlation between variables was assessed using Pearson’s correlation coefficient for normally distributed data and Spearman’s rank correlation coefficient for non-normally distributed data. Specifically, Pearson’s correlation was applied to variables with a normal distribution, including PAQ-C, while Spearman’s rank correlation was used for PACER laps and VO_2_max. The correlation coefficients were interpreted as follows: ‘0.0–0.30’ with no correlation or negligible correlation, ‘0.31–0.50’ low correlation, ‘0.51–0.70’ moderate correlation, ‘0.71–0.9’ high correlation. The statistical significance level was set at 0.05. Post-power analysis was performed using the G*Power 3.1 program. When the effect size was calculated according to the S-index best data, it was found that the effect size of our study was 0.93. With this effect size, it was concluded that 82% power was obtained at a 95% confidence level when there were 20 participants in each group.

## 3. Results

Twenty children diagnosed with asthma and twenty healthy children were included in this study. The groups were similar in terms of age, BMI, and gender (*p* > 0.05, Table 1). All asthmatic participants in our study were using short-acting inhaled bronchodilators.

Participants’ data for dynamic respiratory muscle strength tests are given in Table 2. When the power values were compared between asthmatic (Avg: 1.5 ± 0.9; Best: 4.7 ± 2.5) and healthy (Avg: 2.4 ± 1.3; Best: 6.9 ± 3.3) children, a statistically significant difference was found (*p* = 0.026; *p* = 0.030, respectively). Similarly, there was a statistically significant difference between the S-index data of asthmatic (Avg: 34.1 ± 10.3; Best: 45.6 ± 9.9) and healthy children (Avg: 43.2 ± 12.1; Best: 56.6 ± 14.7) (*p* = 0.015; *p* = 0.008, respectively). The PIF values of children with asthma were significantly lower (Avg: 1.8 ± 0.6; Best: 2.6 ± 0.6) than healthy children (Avg: 2.4 ± 0.7; Best: 3.1 ± 0.9) (*p* = 0.017; *p* = 0.027, respectively). In addition, the mean values of pressure and target load were found to be significantly higher in healthy children than those in the asthmatic group (*p* < 0.05).

Table 3 provides information on PALs and physical fitness levels in both groups. There was no statistically significant difference between the physical fitness and PAL values of the asthmatic children and healthy children groups (*p* > 0.05, Table 3).

**Figure 2:**
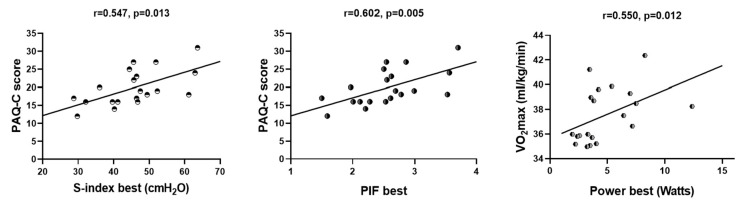
The Relationship Between the S-Index, PIF and PAQ-C, PACER, and VO_2_max in Children with Asthma.

There was a low and a moderate significant positive relationship between theS-index avg (r = 0.498, *p* < 0.025) and S-index best values and the PAQ-C score (r = 0.547, *p* < 0.013). Dynamic inspiratory muscle strength increased and the PAL increased. There was a low and a moderate significant positive correlation between the PIF-avg (r = 0.490, *p* < 0.028) and PIF-best values and the PAQ-C score (r = 0.602, *p* < 0.005) (Table 4, Figure 2).

## 4. Discussion

The present study aimed to compare dynamic respiratory muscle strength parameters between asthmatic children and their healthy peers and examine relationships between dynamic respiratory muscle strength, levels of physical activity, and physical fitness in children with asthma for the first time. The main findings of our study were that peak inspiratory flow and dynamic respiratory muscle strength are impaired in children with asthma compared to healthy peers despite comparable physical activity and physical fitness levels. In addition, higher physical activity scores were observed in asthmatic children as dynamic inspiratory muscle strength and peak inspiratory flow levels increased.

The chronic airflow restrictions resulting from air trapping, increased airway resistance, and lung hyperinflation seen in asthma can alter the position and shape of the diaphragm, change the geometry of the chest wall, and shorten the inspiratory muscles. This affects respiratory muscle performance and pulmonary functions negatively [6,35]. In addition, patients with asthma expend more energy due to the increased workload of breathing. This can lead to respiratory muscle weakness and fatigue [4]. Many studies in the literature have shown that respiratory muscle strength is weaker in children and adults with asthma compared to their healthy peers [6,36,37]. Children and adolescents with asthma have lower PIF values than their healthy peers; this positively correlates with age, height, weight, and respiratory muscle strength [18]. However, in some studies, respiratory muscle strength was measured similarly in children with asthma compared to their healthy peers [15,16]. We found that the dynamic respiratory muscle strength parameters of asthmatic children were significantly lower than healthy children. The 30–40% difference observed in parameters such as S-Index, PIF, and power clearly demonstrates the weakness and limited performance of respiratory muscles. In particular, asthmatic children had a smaller ratio between ‘avg’ and ‘best’ values, suggesting that they have difficulty in reaching maximum performance and optimizing endurance. Decreases in power (Power_avg_, Power_best_) and flow rate (PIF_avg_, PIF_best_) parameters suggest that asthmatic children have less energy production and poorer effective airflow during breathing. These findings support the notion that rehabilitation programs aimed at increasing respiratory muscle strength may contribute to the quality of life of children with asthma by improving both average respiratory muscle performance and maximum capacity [38,39]. Additionally, higher PALs were observed with better S-index and PIF values in the asthmatic child group. Their correlation with increased PA can explain it, with strong respiratory muscles in children allowing PA to be maintained for a longer period, late onset of shortness of breath, and better respiratory endurance. Studies have shown that physical exercise and respiratory muscle training positively affect lung functions in children with asthma [40].

In studies investigating physical fitness in children with asthma, for example, one in which 7731 people between the ages of 6 and 30 years participated, cardiorespiratory endurance, muscle strength, push-ups and sit-ups, and coordination were measured by side jumping and backward balancing. The study showed that cardiorespiratory endurance and muscle strength are not impaired in individuals with asthma [25]. Children with mild to moderate asthma who have exercise-related symptoms exhibit decreased cardiorespiratory fitness, muscle strength, and lung function compared to their healthy peers [24]. Regarding the study conducted by Andrade et al. in Brazil [41], they reported a decline in cardiorespiratory fitness in children with moderate to severe asthma. Berntsen et al. [42] found the opposite in Norway and did not find reduced cardiorespiratory fitness or reduced PAL in individuals with asthma. Cardiopulmonary fitness was generally found to be significantly lower in children with asthma compared to their healthy peers. Decreased VO_2_max was associated with female gender, higher BMI values, and impaired lung function [22]. In our study, the results of the asthmatic group and their healthy peers were similar in terms of physical fitness parameters and cardiovascular endurance running tests. Although, this result supports some findings in the literature, for example, the fact that our asthmatic children with mild to moderate asthma may have comparable physical fitness levels to their healthy peers. In our study, the increase in respiratory power in children with asthma was also highly associated with an increase in VO_2_max levels. Despite lower S-index and PIF results, we did not include any patients with severe and uncontrolled asthma. These could have led to similar fitness levels between groups.

Several studies have explored the relationship between asthma and PALs in children [43,44]. Some studies have found that children with asthma tend to be less physically active compared to those without asthma while others have reported little or no difference [45,46]. When daily PA and shuttle test walking distance were examined between the asthmatic child/adolescent group and their controls, walking distance was lower in asthmatics and the number of steps was similar between the groups but at a low level [47]. Large population studies involving children with asthma of varying severity have generally found similar PALs in both groups [48,49,50]. The studies reporting lower PALs in children with asthma have been small in size or based on questionnaire data. The meta-analysis found no evidence that children and adolescents with and without asthma engaged in different amounts of PA when measured objectively with accelerometers [51]. According to the results of studies in the literature, there are still contradictions regarding the PALs of children with asthma. In our study, the PAL was similar in the groups but was at a low level in accordance with the literature. Mothers of children and adolescents with asthma reported that although they knew that exercise was beneficial for their children, they tended to restrict PA due to various concerns. The most important of these concerns was the fear that their children might become sick during exercise. In addition, factors such as the severity of asthma, the perception of dyspnea at the onset of exercise, and the anxiety associated with it also contribute to these limitations. Although mothers’ concerns may be seen as an attempt to control their children’s health status, they may limit the potential benefits of PA for children with asthma [52].

There are many biological variations that may affect respiratory function, PAL, and physical fitness parameters in children with asthma [3,53]. These variations include factors such as nutritional status, PA habits, hormonal regulation, systemic inflammation, and body composition. It is frequently mentioned in the literature that systemic inflammation can negatively affect muscle strength by increasing muscle fatigue [54,55]. Similarly, deficiencies of nutrients critical for muscle metabolism, such as protein, vitamin D, and magnesium, can both negatively affect muscle performance and trigger systemic inflammation [56,57]. Parents worrying about the severity of their child’s asthma symptoms while exercising can limit PA and lead to weakened respiratory muscles [58]. In this context, future studies that take into account all these biological factors will contribute to a deeper understanding of the effects of asthma on respiratory muscle strength. The main limitation of our study is that we measured PALs with a questionnaire rather than objective methods. Additionally, only children with mild to moderate asthma were included in the asthmatic group, which may introduce selection bias and limit the generalizability of the findings to children with severe asthma. Another potential limitation is our inability to include information on drug dosages, in the analyses, as pharmacological treatment can affect the PAL and pulmonary function. Future studies should aim to use objective methods to measure PALs; include medication information in analyses; and include a more diverse population of children with asthma, including children with severe asthma, to better generalize results. In our study, no molecular and immunological analysis was performed to explain the differences between the groups. Although both groups had similar physical conditions and physical activity levels, future studies should include molecular markers, immune functions, or inflammatory biomarkers to better understand the biological mechanisms underlying these factors.

## 5. Conclusions

In conclusion, our study revealed that although physical activity and fitness levels in children with asthma are similar to their healthy peers, asthmatic children have lower levels of dynamic respiratory muscle strength than their healthy peers. According to the latest GINA guidelines, the ultimate goals of asthma care are to optimize the control of asthma symptoms, maintain lung function as close to normal as possible, and reduce the risk of asthma exacerbations. Although medications can control asthma symptoms, the biomechanical changes caused by asthma require the use of respiratory muscle training in addition to medications to minimize respiratory problems and achieve the best possible outcomes. Since the results of our study showed that dynamic respiratory muscle strength and endurance were particularly impaired, we think that future studies would be useful to determine the effect of rehabilitation programs that include different respiratory muscle training methods and PA counseling in addition to medication on these changes in the respiratory systems of asthmatic individuals. PA and physical fitness, which have a positive contributions to general health in asthma, should be considered and evaluated by health professionals and different strategies like web-based and technology-supported interventions, such as active video gaming for promoting PA and health-related physical fitness parameters, should be applied to improve these parameters by involving families in the process.

## Figures and Tables

**Table 1:** Demographic Information and Respiratory Function.

Variables		Asthmatic Children		Healthy Children		*p* ^a.b^
		X ± SD	Median (IQR)	X ± SD	Median (IQR)	
Age (year)		8.3 ± 1.9	8.5 (6.0–9.5)	8.7 ± 1.8	9.0 (7.0–10.0)	0.528 ^a^
Height (cm)		133.0 ± 0.1	133.0 (128.0–137.0)	135.0 ± 0.1	133.0 (125.0–144.0)	0.058 ^b^
Weight (kg)		31.2 ± 7.7	30.5 (26.0–34.5)	32.2 ± 9.1	34.5 (23.0–39.5)	0.230 ^b^
Body Mass Index (kg/m^2^)		17.5 ± 2.7	17.2 (15.9- 18.6)	17.6 ± 3.5	17.9 (14.3–20.3)	0.066 ^b^
		n (%)		n (%)		
Gender	Girl	10 (50)		12 (60)		0.525 ^c^
	Boy	10 (50)		8 (40)		
Respiratory Function	FVC (L)	1.91 ± 0.43		-		-
	FVC (%)	95.80 ± 8.88				
	FEV_1_ (L)	1.70 ± 0.36				
	FEV_1_ (%)	89.56 ± 6.73				
	PEF	3.25 ± 0.78				
	PEF (%)	81.45 ± 14.96				
	FEV_1_/FVC	89.27 ± 6.73				
	FEV_1_/FVC (%)	104.90 ± 7.90				
	FEF_%25–75_ (L)	2.05 ± 0.47				
	FEF_%25–75_ (%)	90.75 ± 18.81				
Family History		14 (70)		19 (95)		<0.001 ^c^
COVID-19 History		3 (15)		9(45)		0.038 ^c^
Presence of Allergy		13 (65)		0 (0)		<0.001 ^c^
Presence of Rhinitis		5 (25)		1 (5)		0.182 ^c^
Presence of Sinusitis		5 (25)		0 (0)		0.017 ^c^
Presence of Nasal Polyps		2 (10)		1 (5)		1.00 ^c^
Exercise Intolerance		6 (30)		0 (5)		0.020 ^c^
Cold that goes down to the lungs		8 (40)		1 (5)		0.020 ^c^
Cold lasting over ten days		9 (45)		2 (10)		0.031 ^c^
Seasonal Influence		8 (40)		0 (0)		<0.001 ^c^

Statistically significant difference: *p* < 0.05; a: Independent Sample *t*-Test; b: Mann–Whitney U Test; c: Chi-Square Test %: Percentage; X: Mean; SD: Standard Deviation; IQR: Inter Quantile Range; n: Number of Individuals; kg: Kilogram; cm: Centimeter. FVC: Forced Vital Capacity; FEV_1_: Forced Expiratory Volume in One Second; PEF: Peak Expiratory Flow Rate; FEF_%25–75_: Forced Expiratory Flow 25–75%.

**Table 2:** Dynamic Inspiratory Muscle Strength Test Outcomes.

	Asthmatic Children	Healthy Children	*p* ^a.b^
	X ± SD	X ± SD	
S-lndex_avg_ (cmH_2_O)	34.1 ± 10.3	43.2 ± 12.1	0.015 ^a^
S-lndex_best_ (cmH_2_O)	45.6 ± 9.9	56.6 ± 14.7	0.008 ^a^
PIF_avg_ (L/s)	1.8 ± 0.6	2.4 ± 0.7	0.017 ^a^
PIF_best_ (L/s)	2.6 ± 0.6	3.1 ± 0.9	0.027 ^a^
Power_avg_ (Watts)	1.5 ± 0.9	2.4 ± 1.3	0.026 ^a^
Power_best_ (Watts)	4.7 ± 2.5	6.9 ± 3.3	0.030 ^a^
Energy_total_ (Joules)	32.47 (19.1–47.5)	47.3 (24.9–72.9)	0.130 ^b^
Energy_best_ (Joules)	1.27 (0.9–1.9)	2.1 (1.1–2.9)	0.051 ^b^
Target_load_ (cmH_2_O)	16.2 ± 5.4	20.4 ± 5.4	0.018 ^a^
Volume_avg_ (L)	0.9 ± 0.3	1.1 ± 0.3	0.496 ^a^
Volume_best_ (L)	1.3 ± 0.3	1.3 ± 0.3	0.586 ^a^
Flow_avg_ (L/s)	1.2 ± 0.4	1.6 ± 0.6	0.059 ^a^
Flow_best_ (L/s)	2.8 ± 0.6	3.2 ± 0.8	0.061 ^a^
Pressure_avg_ (cmH_2_O)	10.7 ± 3.4	13.4 ± 3.3	0.016 ^a^
Pressure_best_ (cmH_2_O)	24.5 ± 7.8	29.4 ± 8.7	0.069 ^a^

Statistically significant difference: *p* < 0.05; a: Independent Sample *t*-Test; b: Mann–Whitney U Test; X: Mean; SD: Standard Deviation; Avg: Average. PIF: Peak Inspiratory Flow; S-Index: Dynamic Inspiratory Muscle Strength.

**Table 3:** Physical Activity and Physical Fitness Level.

	Asthmatic Children	Healthy Children	*p* ^a.b^
	X ± SD	X ± SD	
Physical Activity			
PAQ-C	2.21 ± 0.54	2.27 ± 0.56	0.708 ^a^
Fıtnessgram			
Sit and reach right	−0.8 ± 6.6	0.2 ± 5.7	0.627 ^a^
Sit and reach left	−0.8 ± 5.7	−0.7 ± 5.2	0.977 ^a^
Trunk lift	18.5 ± 4.6	18.4 ± 4.5	0.972 ^a^
	Median (IQR)	Median (IQR)	
Push-Up	3.0 (1.0–5.5)	3.5 (2.0–6.0)	0.486 ^b^
Curl-Up	8.5 (4.0–20.5)	8.5 (4.0–23.5)	0.957 ^b^
PACER			
Number of Laps	11 (9.0–12.5)	11 (10.0–13.0)	0.504 ^b^
VO_2_max	37.1 (35.7–39.1)	36.5 (35.8–38.8)	0.989 ^b^
ΔHeart Rate	61.5 (48–75)	61.5 (44.5–75)	0.819 ^b^
ΔSpO_2_	0 (−0.5–0)	0 (−0.5–0)	0.962 ^b^
ΔDispne	4 (3–5)	3.5 (2–4)	0.161 ^b^
ΔFatigue	3 (1.5–4)	3 (1–4)	0.433 ^b^
ΔLeg Fatigue	2 (1–4)	1.5 (1–3)	0.334 ^b^

Statistically significant difference: *p* < 0.05; a: Independent Sample *t*-Test; b: Mann–Whitney U Test; X: Mean; SD: Standard Deviation; IQR: Inter Quantile Range; PAQ-C: Physical Activity Questionnaire for Older Children; PACER: Progressive Aerobic Cardiovascular Endurance Run; VO_2_max: Maximum Oxygen Consumption; SpO_2_: Oxygen Saturation.

**Table 4:** The Relationship Between the S-Index, PIF and PAQ-C, PACER, and VO_2_max in Children with Asthma.

	PAQ-C Score	PACER Number of Laps (n)	VO_2_max
S-index_avg_	r^a^ = 0.498; *p* = 0.025 *	r^b^ = 0.024; *p* = 0.922	r^b^ = 0.320; *p* = 0.168
S-index_best_	r^a^ = 0.547; *p* = 0.013 *	r^b^ = −0.114; *p* = 0.633	r^b^ = 0.237; *p* = 0.315
PIF_avg_	r^a^ = 0.490; *p* = 0.028 *	r^b^ = 0.001; *p* = 0.997	r^b^ = 0.378; *p* = 0.101
PIF_best_	r^a^ = 0.602; *p* = 0.005 *	r^b^ = −0.102; *p* = 0.670	r^b^ = 0.239; *p* = 0.310
Power_avg_	r^a^ = 0.348; *p* = 0.132	r^b^ = −0.069; *p* = 0.072	r^b^ = 0.387; *p* = 0.092
Power_best_	r^a^ = 0.355; *p* = 0.124	r^b^ = 0.072; *p* = 0.763	r^b^ = 0.550; *p* = 0.012 *

Statistically significant difference; *: *p* < 0.05; r^a^: Pearson’s correlation coefficient; r^b^: Spearman’s rank correlation; −: negative correlation; Avg: Average; PIF: Peak İnspiratory Flow; S-Index: Dynamic Inspiratory Muscle Strength; PAQ-C: Physical Activity Questionnaire for Older Children; PACER: Progressive Aerobic Cardiovascular Endurance Run; VO_2_max: Maximum Oxygen Consumption.

## Data Availability

The data that support the findings of this study are available from the corresponding author [S.N.O.], upon reasonable request.
